# Saccadic Oscillations After Donepezil Administration in Patients with Dementia: Time Course and Clinical Correlates

**DOI:** 10.3390/jemr19050094

**Published:** 2026-08-27

**Authors:** Ileok Jung, Ji-Soo Kim, Moon-Ho Park

**Affiliations:** 1 Department of Neurology, Konkuk University Medical Center, Seoul 05030, Republic of Korea; ileokjung@kuh.ac.kr; 2 Dizziness Center, Clinical Neuroscience Center, Department of Neurology, Seoul National University Bundang Hospital, Seongnam 13620, Republic of Korea; jisookim@snu.ac.kr; 3 Department of Neurology, Seoul National University Bundang Hospital, Seoul National University College of Medicine, Seongnam 13620, Republic of Korea; 4 Department of Neurology, Korea University Ansan Hospital, Korea University College of Medicine, Ansan 15355, Republic of Korea

**Keywords:** saccadic oscillations, square-wave jerks, donepezil, dementia, dizziness

## Abstract

Donepezil, a cholinesterase inhibitor widely used in dementia, can be associated with saccadic oscillations (SO), but their time course and clinical relevance are unclear. In this prospective observational study, 18 patients with dementia underwent video-oculography at baseline (V0), one month (V1), and three months (V2) after initiation or dose escalation of donepezil. SO were counted over a single 30 s recording during visual fixation and in darkness, expressed as events/30 s. Dizziness was assessed with the Dizziness Handicap Inventory (DHI) and the Vestibular Disorders Activities of Daily Living (VADL) scale. Timepoints were compared using the Wilcoxon signed-rank test, and within-patient associations by repeated-measures correlation (rmcorr). SO with fixation increased from a median of 0.0 (interquartile range 0.0–1.0) events/30 s at V0 to 13.5 (7.2–15.8) at V1 (unadjusted *p* = 0.008, adjusted *p* = 0.023), then declined, but the difference from baseline lost statistical significance after multiple-comparison adjustment at V2 (6.5 [0.0–11.5]; unadjusted *p* = 0.031, adjusted *p* = 0.063). SO without fixation did not change (all *p* > 0.12). On rmcorr, SO without fixation was associated with the VADL (r = 0.59, *p* = 0.005) but neither measure was associated with the DHI. A transient, fixation-specific increase in SO was observed, peaking at one month and only partially resolved by three months; it correlated with functional disability rather than subjective handicap, suggesting that video-oculography warrants further study as a potential pharmacodynamic correlate of cholinergic drug effect in dementia.

## 1. Introduction

**Figure 1:**
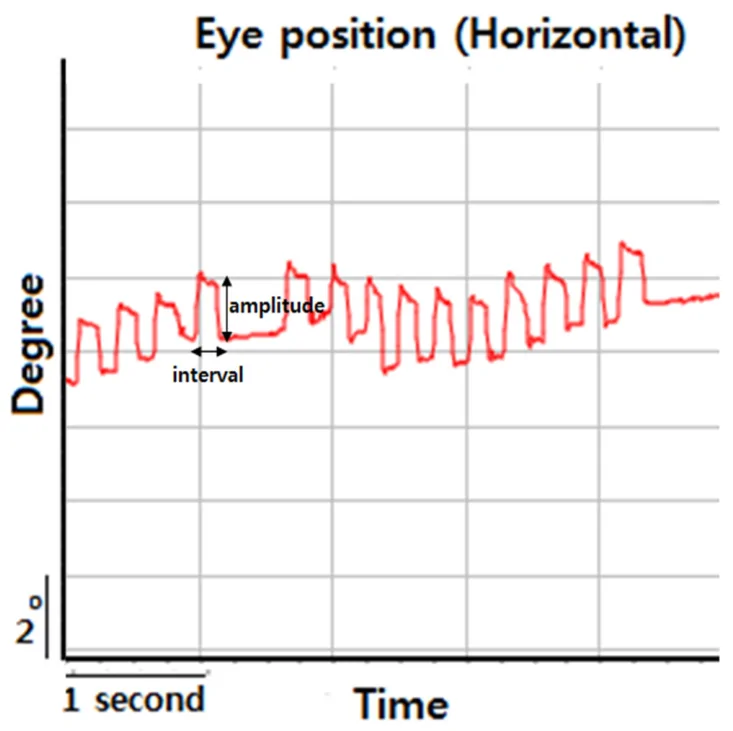
Definition of square-wave jerks.

Donepezil, a reversible acetylcholinesterase inhibitor (AChEI), is widely prescribed for treating Alzheimer’s disease and vascular dementia [1]. By inhibiting acetylcholine (ACh) breakdown, it enhances cholinergic neurotransmission, but frequently induces adverse effects, including dizziness as a major contributor to poor medication adherence as well as gastrointestinal symptoms or bradycardia [1,2]. Dizziness occurs in 6–8% of patients on standard doses (5–10 mg/day) and increases at higher doses (23 mg/day), with most discontinuations due to adverse effects occurring within the first month of treatment [2]. Despite its prevalence, the pathophysiological mechanisms of dizziness associated with donepezil remain poorly understood, and objective indicators for this symptom are notably lacking [2,3]. Recent observation suggests that some patients report dizziness and unsteadiness specifically after the initiation or dose escalation of donepezil, accompanied by frequent saccadic oscillations (SO) [4]. SO are sustained involuntary, conjugate fast eye movements away from the target [5]. SO encompass a spectrum of fixation instabilities including square-wave jerks (SWJs), macro square-wave jerks, and macro saccadic oscillations [6,7,8]. These movements disrupt stable vision and can cause oscillopsia that may contribute to the sensation of dizziness [5,6]. SWJs are the most common type of saccadic intrusions, and consist of small conjugate couplets of horizontal back-to-back saccades ranging from 0.5 to 5 degrees, taking the eye away from the fixation point and then returning it after a normal length intersaccadic interval of about 200 ms (Figure 1) [9]. Frequent SWJs are also called square-wave oscillations (9–16 per minute, or >20 per minute in darkness) [9].

Even though the mechanism of SO is unclear, hypotheses have been built on the model of saccade generation that involves the frontal eye fields and superior colliculus (SC) [6,10]. The omnipause neuron (OPN) has also been considered the pathophysiological basis of opsoclonus and ocular flutter [11]. The neural network that generates saccades is potentially unstable due to positive feedback as a consequence of the microcircuitry of the excitatory and inhibitory burst neurons [12]. Either an increase in neural excitability or a reduced inhibition of the OPN can cause instability and oscillations [12,13]. Relatively small drug-induced changes in the synaptic weighting of such circuits could therefore produce or suppress SO [14]. The superior colliculus (SC), a central structure for saccadic control, contains dense cholinergic innervation in its layers [15,16]. Cholinergic inputs from the pedunculopontine tegmental nucleus (PPTg) and parabigeminal nucleus (PBN) to the SC play a critical facilitatory role in saccade initiation and visual signal processing [16,17,18]. Sudden increases in ACh concentration may overstimulate the nicotinic and muscarinic receptors within these circuits, triggering involuntary SO [17]. Specifically, the nicotinic acetylcholine receptors mediate fast excitatory responses in SC neurons, while muscarinic receptors modulate neuronal excitability through both excitatory and inhibitory mechanisms [15,19]. While preliminary case series have identified that dizziness associated with donepezil was accompanied by frequent SO that improve after drug discontinuation, robust longitudinal data are required to confirm its clinical significance [4]. In this prospective study, we used serial video-oculography (VOG) to characterize the time course of SO in patients with dementia following donepezil initiation or dose escalation. Our objective was to characterize the time course of SO following donepezil and to explore their relationship with dizziness-related outcomes in patients with dementia.

## 2. Methods

### 2.1. Study Design and Participants

This was a prospective, longitudinal observational study conducted at the Department of Neurology, Korea University Ansan Hospital. We analyzed adult patients (aged ≥18 years) diagnosed with mild cognitive impairment or mild-to-moderate Alzheimer’s disease/vascular dementia, based on the Clinical Dementia Rating (CDR) 0.5–2 or Global Deterioration Scale (GDS) 3–6. Inclusion criteria required patients to be either treatment-naïve and starting donepezil or scheduled for a dose increment. Exclusion criteria included: (1) occurrence of vestibulopathy within one month; (2) use of vestibulotoxic or neurotropic medications; and (3) presence of severe systemic disease.

### 2.2. Ethics Approvals and Trial Registration

The study followed the Declaration of Helsinki and was approved by the Institutional Review Board (No. 2019AS0020) and this prospective cohort study was retrospectively registered at the Clinical Research Information Service (CRIS; KCT0009211). Written informed consent was obtained from all participants or their legal representatives.

### 2.3. Assessments and VOG Protocol

Evaluations were performed at baseline (V0), one month (V1), and three months (V2) after initiation or dose escalation of donepezil. The severity of dizziness was assessed using the Dizziness Handicap Inventory (DHI) and the Vestibular Disorders Activities of Daily Living (VADL) scale. Functional status and mood were monitored using the Beck Depression Inventory (BDI). Binocular horizontal eye movements were recorded using infrared reflection oculography (SLMED, Seoul, Korea). The VOG protocol consisted of the following steps: (1) The patient was instructed to fixate on a target located 1.2 m away, and eye movements were recorded for 30 s. During the recording, patients were encouraged to keep their eyes as still as possible and maintain continuous fixation on the target. (2) Fixation was then removed by covering both eyes, and eye movements were recorded in complete darkness for 30 s, with patients instructed to keep their eyes as still as possible and look straight ahead. During the procedures described in (1) and (2), recordings were reviewed for waveforms composed of two conjugate saccades separated by a brief intersaccadic interval. SO included SWJs and macro-SWJs while isolated saccadic pulses were excluded to differentiate SO from eye drift. Video recordings were reviewed to exclude artifacts related to blinking-related eye movements. The frequency of SO was counted over the 30 s recording in each condition (fixation and non-fixation) and expressed as events per 30 s. SWJ durations were determined from the interval between the onset of the error-producing saccade and completion of the error-correcting saccade (Figure 1). Mean SWJ durations were determined from 10 to 30 samples in each patient.

Representative horizontal eye-position recording demonstrating a square-wave jerk (SWJ). The amplitude and intersaccadic interval of the SWJ are indicated in the figure.

### 2.4. Statistical Analysis

Normality of continuous variables was assessed using the Shapiro–Wilk test. As the variables violated the normality assumption and were heavily zero-inflated, data are summarized as median with interquartile range (IQR), and non-parametric methods were used throughout. This was an exploratory pilot study using a convenience sample of eligible patients at a single center; no a priori sample-size calculation was performed. A mixed-effects model, which would use all available observations simultaneously, was considered but not adopted, because the small sample and heavy zero-inflation of the SO counts make such models prone to convergence problems and overfitting; we therefore used non-parametric available-case analyses and report the number of available pairs for each contrast.

Changes in SO frequency and clinical scale scores between timepoints (V0 vs. V1, V1 vs. V2, and V0 vs. V2) were compared using the Wilcoxon signed-rank test on all available paired observations for each contrast. Because attrition differed across timepoints, the number of available pairs varies between contrasts and is reported with each result. To account for multiple pairwise testing, *p*-values were adjusted within each outcome using the Holm–Bonferroni method. The overall effect of time across the three timepoints was additionally assessed with the Friedman test in the subset of patients with complete data at all timepoints.

The association between SO frequency and dizziness-related outcomes (DHI and VADL) was assessed using repeated-measures correlation (rmcorr), which estimates the common within-participant association across repeated observations while accounting for the non-independence of measurements from the same individual. Rmcorr coefficients are reported with 95% confidence intervals. Missing data were handled by available-case analysis under a missing-at-random assumption, without imputation; one non-fixation recording that could not be quantified because of concurrent nystagmus was treated as missing rather than zero.

A two-sided *p*-value < 0.05 was considered statistically significant. Analyses were performed in Python 3.10 (SciPy) and R 4.1.0 (rmcorr package).

## 3. Results

### 3.1. Participants and Follow-Up

Eighteen patients (mean age 70.3 ± 11.8 years; 11 women) were enrolled. The cohort had mild-to-moderate cognitive impairment (mean Mini-Mental State Examination (MMSE) of 22.1 ± 3.9; mean GDS 3.4 ± 1.0). The baseline dizziness burden was variable and often mild [DHI median 13.0 (IQR 4.5–46.0); VADL median 39.5 (IQR 29.8–72.2)] (Table 1).

SO-with-fixation data were available for 17 patients at V0, 14 at V1, and 10 at V2; corresponding numbers for SO-without-fixation were 17, 13, and 10, reflecting progressive attrition and one non-assessable recording.

Patients who completed the three-month visit (*n* = 10) did not differ significantly from those who did not (*n* = 8) in age, MMSE, or GDS (all *p* > 0.24). Baseline DHI tended to be higher among non-completers [median 29.0 (IQR 12.5–73.0)] than completers [median 9.0 (IQR 2.0–21.0); *p* = 0.091], raising the possibility that attrition was related to baseline dizziness severity; the potential impact of this informative dropout on the robustness of the results is considered in the Limitations.

### 3.2. Saccadic Oscillations After Donepezil Initiation or Dose Escalation (Table 2)

SO with fixation increased markedly one month after donepezil initiation (5 mg) or dose escalation (5 mg → 10 mg; V1). The median frequency rose from 0.0 (IQR 0.0–1.0) events/30 s at baseline to 13.5 (IQR 7.2–15.8) events/30 s at V1 (Wilcoxon signed-rank, *n* = 14, *p* = 0.008). In contrast, SO without fixation did not change significantly [V0: 0.0 (IQR 0.0–15.0) vs. V1: 0.0 (IQR 0.0–15.0) events/30 s; *n* = 13, *p* = 0.812].

Subjective dizziness [DHI: 13.0 (IQR 4.5–46.0) at V0 vs. 6.0 (IQR 4.0–22.0) at V1; *n* = 15, *p* = 0.528] and the functional and psychological scales (VADL, BDI) did not change significantly at V1 (Table 2).

**Table 2:** Serial outcomes across timepoints and paired comparisons.

Outcome (Events/30 s or Score)	V0	V1	V2	V0–V1 *p* (Adj.)	V1–V2 *p* (Adj.)	V0–V2 *p* (Adj.)
SO with fixation	0.0 (0.0–1.0)	13.5 (7.2–15.8)	6.5 (0.0–11.5)	0.008 (0.023)	0.438 (0.438)	0.031 (0.063)
SO without fixation	0.0 (0.0–15.0)	0.0 (0.0–15.0)	1.5 (0.0–13.0)	0.812 (0.812)	0.125 (0.375)	0.312 (0.625)
DHI	13.0 (4.5–46.0)	6.0 (4.0–22.0)	8.0 (0.0–46.0)	0.528 (1.000)	0.922 (1.000)	0.760 (1.000)
VADL	39.5 (29.8–72.2)	45.0 (36.5–70.5)	32.5 (26.0–67.5)	0.245 (0.490)	0.106 (0.316)	0.456 (0.490)
BDI	8.5 (5.2–13.2)	10.0 (4.0–13.0)	9.0 (6.5–19.2)	0.207 (0.586)	0.195 (0.586)	0.500 (0.586)

Values are presented as median (interquartile range). Pairwise comparisons between timepoints were performed using the Wilcoxon signed-rank test on all available paired observations for each contrast; because of follow-up attrition, the number of available pairs differed across contrasts. Available pairs (V0–V1/V1–V2/V0–V2): SO with fixation, 14/8/10; SO without fixation, 13/8/10; DHI, 15/12/14; VADL, 15/12/14; BDI, 9/10/8. SO, saccadic oscillations; DHI, dizziness handicap inventory; VADL, Vestibular Disorders Activities of Daily Living scale; BDI, Beck depression inventory. *p* < 0.05 was considered statistically significant. *p*-values are shown as unadjusted (Holm-adjusted within each outcome across the three contrasts). The V0–V1 increase in SO with fixation remained significant after adjustment (adjusted *p* = 0.023); the V0–V2 difference was significant only before adjustment.

### 3.3. Time Course over Three Months

Among the eight patients with complete data at all three timepoints, the Friedman test did not identify a significant overall effect of time on SO with fixation (χ^2^ = 4.16, *p* = 0.125), reflecting the substantial loss of power in this small complete-case subset. The principal finding—the increase in SO with fixation from V0 to V1—was significant in the larger available-pair sample [*n* = 14, *p* = 0.008; Holm-adjusted *p* = 0.023]. At three months, SO with fixation had declined from its one-month peak; it was higher than baseline before correction but this difference did not survive multiple-comparison adjustment [V0: 0.0 (IQR 0.0–1.0) vs. V2: 6.5 (IQR 0.0–11.5) events/30 s; *n* = 10, unadjusted *p* = 0.031, Holm-adjusted *p* = 0.063]. The reduction from V1 to V2 was not statistically significant [V1: 14.0 (IQR 5.2–17.0) vs. V2: 6.5 (IQR 0.0–11.5) events/30 s; *n* = 8, *p* = 0.438]. SO without fixation showed no significant change across any contrast (all *p* > 0.12). Because these contrasts are based on different, partly overlapping subsets of patients, effect estimates should not be directly compared across timepoints.

### 3.4. Correlation Between SO and Dizziness-Related Outcomes

In exploratory rmcorr analyses, which were not pre-specified and were not adjusted for multiple comparisons, SO without fixation was positively associated with VADL (r = 0.59, 95% CI 0.21 to 0.81; *p* = 0.005). SO without fixation and DHI (r = 0.42, 95% CI −0.01 to 0.72; *p* = 0.055) and SO with fixation and VADL (r = 0.40, 95% CI −0.03 to 0.70; *p* = 0.066) showed positive associations of borderline significance, whereas SO with fixation and DHI were not associated (r = −0.19; *p* = 0.410) (Table 3).

Taken together, a significant increase in SO with fixation was observed at one month following donepezil administration that was only partially resolved by three months, and within-patient increases in SO without fixation were accompanied by greater functional disability on the VADL.

## 4. Discussion

This study demonstrates that initiation or dose escalation of donepezil is associated with a transient increase in SO with fixation in patients with dementia. SO with fixation following donepezil initiation or dose escalation increased significantly at one month and declined only partially by three months, despite continued medication. In contrast, SO without fixation showed no significant changes following donepezil initiation or escalation. Notably, the increase in SO with fixation was not associated with subjective dizziness handicap as measured by DHI. In an exploratory, post-hoc analysis, within-patient increases in SO without fixation showed a positive association with functional disability as measured by the VADL.

Medication of donepezil is usually initiated at 5 mg a day, and increased over several weeks to 10 mg per day [20]. The maximum daily dose of donepezil is 23 mg. The higher dose increases the incidence of cholinergic side effects [21]. Common adverse effects include gastrointestinal symptoms—nausea, diarrhea, anorexia, and abdominal pain as well as dizziness or bradycardia [20]. AChE inhibitors enhance the release of acetylcholine via a nicotinic mechanism. Most studies on the AChE inhibitors have focused on cortical regions including the hippocampus and prefrontal cortex. However, it is well established that the superficial and intermediate gray layers of the SC have dense cholinergic innervation across mammalian species [22,23].

**Figure 2:**
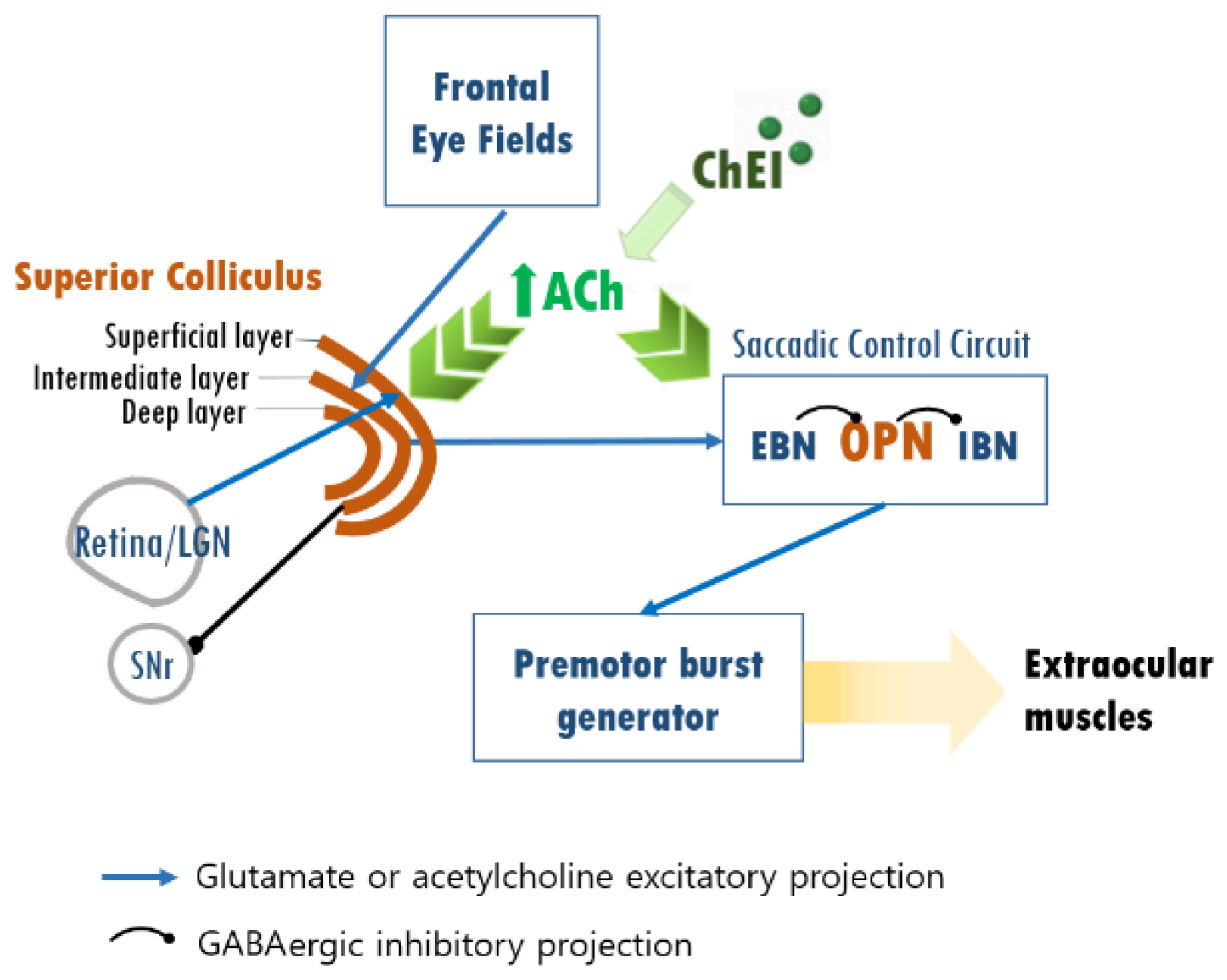
Hypothesized mechanism underlying the association between donepezil exposure and saccadic oscillations. Abbreviations: ACh, acetylcholine; ChEI, cholinesterase inhibitor; EBN, excitatory burst neuron; IBN, inhibitory burst neuron; LGN, lateral geniculate nucleus; OPN, omnipause neuron; SC, superior colliculus; SNr, substantia nigra pars reticulata. Arrows: Blue arrows, glutamatergic or cholinergic excitatory projections; filled circles on black lines, GABAergic inhibitory projections; green arrows, cholinergic enhancement by ChEI.

Cholinergic inputs to the intermediate layer of the SC facilitate the generation of motor outputs involved in saccade initiation. In an animal study, nicotine injection into the intermediate and deep layers of the SC abruptly increases the occurrences of express saccades [24]. In addition, experimental studies have demonstrated that cholinergic activation within the SC modulates inhibitory gamma-aminobutyric acid (GABA) circuitry involved in saccadic control [25]. These findings suggest that activation of nicotinic ACh receptors within the SC facilitates saccadic initiation and may alter the balance between excitatory and inhibitory oculomotor networks [26,27]. Furthermore, cholinergic inputs have been reported to suppress GABAergic synaptic transmission in SC neurons through presynaptic muscarinic receptor activation [28]. Such cholinergic modulation of inhibitory circuitry could destabilize the saccadic system. Consequently, increased synaptic ACh concentrations induced by ChEIs may reduce GABAergic inhibition within the SC, thereby contributing to the generation of SO (Figure 2).

Donepezil inhibits acetylcholinesterase, increasing synaptic acetylcholine (ACh) within the saccadic control network. During visual fixation, excitatory inputs from the retina/lateral geniculate nucleus and frontal eye fields activate the superior colliculus (SC), which is further excited by elevated ACh, while the substantia nigra pars reticulata provides tonic GABAergic inhibition to the SC. Excess cholinergic activity suppresses GABAergic inhibition within the SC through presynaptic muscarinic receptor activation, reducing inhibitory control over the saccadic burst circuitry. This imbalance destabilizes the omnipause neuron (OPN)-burst neuron network, in which OPNs normally maintain tonic inhibition over burst neurons during fixation. Transient failure of this OPN-mediated suppression allows inappropriate activation of the premotor burst generator and the extraocular muscles, producing involuntary saccadic oscillations.

### 4.1. Neural Circuit Mechanism and Temporal Dynamics

The observed increase in SO after initiation or dose escalation of donepezil likely reflects transient instability within the saccadic control network. These networks involve interactions among the brainstem, cerebellum, and superior colliculus, which together regulate fixation and saccadic initiation [8,9,16,18].

Enhanced cholinergic activity may increase excitability within these circuits and lower the threshold for involuntary SO. We interpret the fixation-specific increase as follows, distinguishing established experimental evidence from our hypothesis. Experimental evidence indicates that the SC receives dense cholinergic innervation, that cholinergic activation facilitates saccade initiation, and that cholinergic inputs suppress GABAergic transmission in SC neurons [24,25,28]. On this basis, we hypothesize that the acute rise in acetylcholine produced by donepezil reduces GABAergic inhibition within the SC and destabilizes the balance between saccadic burst neurons and omnipause neurons (OPNs), transiently releasing involuntary SO. Although SO with fixation declined from its one-month peak, it had not returned to baseline by three months, suggesting that any adaptive rebalancing was, at most, partial over this interval.

Supranuclear inputs from the SC and striate cortex are known to influence pause cell activity [26] and SWJs result from unwanted supranuclear trigger signals that interrupt pause cell activity, thereby releasing saccadic burst units [5,27]. Rather than indicating structural dysfunction, the increase of SO in our cohort appears to represent a functional disturbance of the oculomotor control [16,28]. The mechanisms underlying SO generation involve dysfunction of the OPNs, which normally provide tonic inhibition over the saccadic burst neurons in the paramedian pontine reticular formation. Excessive cholinergic stimulation may transiently impair this inhibitory control, allowing spontaneous burst neuron activity and resultant fixation instabilities [6,29,30,31]. The stability of saccadic velocity despite the increase in SO frequency may indicate that the observed effect is not accompanied by a generalized impairment of saccade generation; however, the specific neural substrates cannot be determined from the present data [32,33].

The more prominent increase in SO during fixation may reflect instability within the fixation-maintaining network centered on the SC and OPN. Experimental studies have shown that the rostral SC provides monosynaptic excitatory input to OPNs, which maintain tonic firing during visual fixation and suppress unwanted saccades [33].

Because fixation requires sustained activation of the rostral SC–OPN network, we hypothesize that excessive cholinergic enhancement destabilizes this network preferentially during fixation, making fixation conditions more susceptible to SO than non-fixation states. This interpretation is consistent with, but not proven by, our observation that the drug-related increase was confined to the fixation condition.

The time course—a one-month peak followed by an incomplete decline—may reflect adaptive receptor desensitization during sustained cholinergic stimulation, a process described for chronic acetylcholinesterase inhibition, although this remains speculative in the present context and was not directly assessed [34,35].

Taken together, these findings are consistent with donepezil-associated modulation of saccadic control circuits that is at least partially reversible over three months, rather than a fixed structural impairment.

### 4.2. Clinical Significance of SO Following Donepezil Initiation or Dose Escalation

The clinical relevance of the observed SO is clarified by how it relates to different dizziness-related measures. Whereas the temporal increase observed following donepezil administration was specific to SO recorded during visual fixation, the within-patient association with functional disability was strongest for SO recorded without fixation (VADL: r = 0.59, *p* = 0.005), with a borderline association for SO with fixation (r = 0.40, *p* = 0.066). By contrast, neither SO measure was significantly associated with the DHI. Although exploratory and post-hoc, this finding raises the possibility that SO may be more closely related to functional measures such as the VADL than to subjective dizziness handicap as measured by the DHI. The DHI emphasizes perceived emotional and situational handicap from dizziness [36], whereas the VADL measures functional independence in daily activities [37]; the multifactorial nature of subjective dizziness—which integrates vestibular, proprioceptive, and central processing beyond the saccadic system—may explain why oculomotor instability relates more closely to measurable functional impact than to perceived handicap. This distinction may be particularly relevant in patients with dementia. Although both instruments are self-reported, they differ in cognitive demand: the VADL asks patients to rate difficulty with concrete, observable daily activities, whereas the DHI requires a more abstract appraisal of the emotional and situational handicap imposed by dizziness [36]. Self-appraisal of this kind is often compromised by impaired self-awareness and interoception in cognitive impairment [38], which may render the DHI less sensitive than the VADL in this population. The association of SO with VADL but not DHI may thus reflect, at least in part, differences in the types of information captured by these instruments.

Nevertheless, the clinical utility of VOG-based SO monitoring should not be dismissed. First, because the present study did not compare treatment continuation with discontinuation, its data cannot guide management decisions; whether patients who develop SO can be safely maintained on therapy rather than discontinued is a hypothesis that requires prospective, controlled evaluation. Second, in patients with cognitive impairment who may have difficulty accurately reporting symptoms, VOG-based SO measurement provides an objective and quantifiable correlate of cholinergic drug effect. Third, identification of risk factors such as white matter hyperintensities could enable pre-treatment risk stratification and personalized dosing strategies. In terms of feasibility, video-oculography is non-invasive and rapid, requiring under a minute of recording per condition, and portable video-goggle systems are increasingly available in neurology and neuro-otology clinics; however, cost, the need for trained interpretation, and the absence of automated SO detection in routine devices currently limit widespread use, and simplified, ideally automated, protocols would be needed before routine adoption.

Not all patients developed frequent SO, and this suggests an individual variation in vulnerability. White matter hyperintensities (leukoaraiosis) represent a potential risk factor for oculomotor dysfunction in elderly patients [39]. Cerebral white matter changes are associated with various oculomotor abnormalities including impaired fixation stability and increased saccadic intrusions [27,39,40]. In patients with small vessel cerebrovascular disease, oculomotor deficits correlate with both white matter lesion load and cognitive dysfunction, suggesting that disruption of frontal–subcortical pathways may lower the threshold for saccadic circuit instability [7,39,40]. In elderly patients with pre-existing white matter hyperintensities or cortical atrophy, the saccadic instability may be more prominent, making them more susceptible to the side effects of cholinesterase inhibitors [8]. Furthermore, individual variations in cholinergic receptor subtypes, baseline receptor density, and genetic polymorphisms affecting acetylcholine metabolism may also contribute to differential susceptibility to SO following donepezil initiation or dose escalation [35,41].

## 5. Limitations and Conclusions

This study has several limitations. First, this was an exploratory pilot study with a small convenience sample (*n* = 18) and no a priori power calculation; the findings are hypothesis-generating and are interpreted through effect sizes and confidence intervals rather than as confirmatory results. Second, follow-up attrition was substantial, and only 10 of 18 patients completed the three-month visit. Although completers and non-completers were similar in age, cognition, and baseline SO, baseline DHI tended to be higher among non-completers (29.0 vs. 9.0; *p* = 0.091), raising the possibility of informative dropout; the three-month estimates therefore derive from a smaller and possibly less symptomatic subgroup and should be interpreted with corresponding caution. Because pairwise contrasts were based on different, partly overlapping subsets, effect estimates should not be directly compared across timepoints. Third, the study lacked a non-treated control group, so we cannot exclude time- or disease-related changes unrelated to donepezil; a controlled design is required before any pharmacodynamic-marker role can be established. Fourth, the correlations between SO and dizziness-related outcomes were post-hoc, were not adjusted for multiple testing, and had wide confidence intervals (e.g., SO without fixation vs. VADL, r = 0.59, 95% CI 0.21–0.81); they should be regarded as hypothesis-generating. Because dizziness outcomes relied on patient self-report, the reduced reliability of subjective appraisal in cognitively impaired individuals may also have attenuated the association between SO and the DHI. Finally, we did not systematically assess white matter lesion burden, and quantification of SO in darkness is inherently more challenging in the absence of a fixation target, which may introduce measurement variability.

Future studies with larger sample sizes, longer follow-up periods, and comprehensive neuroimaging assessment are needed to validate these findings and to determine whether SO monitoring can guide personalized donepezil dosing strategies.

Despite these limitations, this study demonstrates that donepezil initiation or dose escalation is associated with a transient increase in SO during visual fixation, peaking at one month and declining only partially by three months. The observed SO was not associated with subjective dizziness handicap (DHI). In an exploratory, post-hoc analysis, SO without fixation showed a positive within-patient association with functional disability (VADL); this analysis was not pre-specified and was not adjusted for multiple comparisons, and the finding requires confirmation. The observed SO thus appears to be a quantifiable, partially reversible oculomotor phenomenon. Whether SO is genuinely associated with functional disability and whether it can serve as an objective pharmacodynamic correlate of cholinergic drug effects require confirmation in larger, controlled studies. Future research should examine the relationship between SO, white matter lesion burden, and long-term clinical outcomes, and whether SO monitoring could inform personalized dosing of cholinesterase inhibitors.

## Figures and Tables

**Table 1:** Baseline Demographic and Clinical Characteristics.

Demographic Characteristics (N = 18)		
Sex (woman %)		11 (61.1%)
Age (years)		70.3 ± 11.8
Cognitive Function	MMSE score	22.1 ± 3.9
	GDS	3.4 ± 1.0

Values are presented as *n* (%) or mean ± standard deviation. MMSE, Mini-Mental State Examination; GDS, Global Deterioration Scale.

**Table 3:** Repeated-measures correlation of SO frequency with DHI and VADL.

Pair	r (rmcorr)	95% CI	Observations	*p*
SO with fixation—DHI	−0.19	−0.56 to 0.26	38	0.410
SO with fixation—VADL	0.40	−0.03 to 0.70	38	0.066
SO without fixation—DHI	0.42	−0.01 to 0.72	37	0.055
SO without fixation—VADL	0.59	0.21 to 0.81	37	0.005

SO, saccadic oscillations; DHI, dizziness handicap inventory; VADL, Vestibular Disorders Activities of Daily Living scale; rmcorr, repeated-measures correlation; CI, confidence interval. Rmcorr estimates the common within-participant association across repeated observations, accounting for non-independence of measurements from the same individual. *p* values < 0.05 were considered statistically significant.

## Data Availability

The original contributions presented in this study are included in the article. Further inquiries can be directed to the corresponding authors.
